# Secondary hemophagocytic lymphohistiocytosis in a 5-month-old infant with IBD post-COVID-19: a case report

**DOI:** 10.3389/fped.2025.1536066

**Published:** 2025-05-01

**Authors:** Yulin Chen, Xiaoli He, Deyuan Li, Lina Qiao, Guoyan Lu

**Affiliations:** Department of Pediatrics, Ministry of Education Key Laboratory of Women and Children’s Diseases and Birth Defects, West China Second University Hospital, Sichuan University, Chengdu, Sichuan, China

**Keywords:** inflammatory bowel diseases, hemophagocytic lymphohistiocytosis, IL-10RA, COVID-19, child

## Abstract

**Background:**

COVID-19 is known to induce cytokine storms and inappropriate cytotoxic immune responses. Hemophagocytic lymphohistiocytosis (HLH) is an underrecognized condition due to a hyperinflammatory syndrome characterized by fulminant hypercytokinemia with a high mortality burden. Cases of severe acute respiratory syndrome coronavirus 2 (SARS-CoV-2) induced secondary HLH during and post-infection have been sparsely reported in children. Inflammatory bowel disease (IBD) is a chronic, relapsing disorder of the gastrointestinal tract with a multifactorial etiology involving genetic, environmental, and immunological factors. To date, secondary HLH associated with COVID-19 in very early-onset inflammatory bowel disease (VEO-IBD) has not been reported. This case report aims to enhance understanding of the clinical manifestations of VEO-IBD and HLH, thereby facilitating the timely diagnosis and management of this rare condition.

**Case presentation:**

We present the case of a 5-month-old Chinese female infant diagnosed with HLH following COVID-19 infection. The patient presented with hemophagocytic syndrome, which included recurrent fever, hepatosplenomegaly, cytopenia, hyperferritinemia, hypertriglyceridemia, and hypofibrinogenemia, after exposure to her mother, who had been diagnosed with COVID-19. Whole-exome sequencing(WES) identified heterozygous mutations in the IL-10RA gene: c.537 G > A (inherited from her mother) and c.301 C > T (inherited from her father), who was ultimately identified as having VEO-IBD. Despite receiving nutritional support, intravenous immunoglobulin (IVIG), and dexamethasone therapy, the patient continued to experience anemia, diarrhea, and refractory gastrointestinal bleeding. Following a brief improvement after interventional treatment, the parents declined further medical interventions, signed for discharge, and the infant sadly passed away three months later.

**Conclusion:**

Rare genetic variants play a pivotal role in the pathogenesis of VEO-IBD, particularly in infants diagnosed before the age of one. These cases often demonstrate resistance to various immunosuppressive therapies and have a poor prognosis with conventional treatments. Our findings highlight the potential increased risk of severe HLH in patients with VEO-IBD and concurrent COVID-19, underscoring the need for comprehensive and vigilant differential diagnosis when patients exhibit symptoms suggestive of multi-organ damage.

## Introduction

IBD can occur at any age and is often associated with monogenic defects or primary immune deficiencies. VEO-IBD is defined as IBD diagnosed in children younger than six years of age (1), accounting for approximately 15% of pediatric IBD cases, with 1% of these cases presenting before the age of one (2). Children with VEO-IBD frequently experience severe gastrointestinal symptoms, including intractable colitis with hematochezia, perianal abscesses, and fistulae (3). According to the OMIM database, IBD is classified into 31 subtypes based on distinct pathogenic genes or chromosomal regions. Among Chinese IBD patients, subtypes 25(IL10RB gene) and 28(IL10RA gene) are most common (4), with IBD type 28 inherited in an autosomal recessive manner.

HLH is a rare, rapidly progressing, immune-mediated life-threatening disease characterized by an uncontrolled and ineffective immune response, often triggered by infectious agents. Secondary HLH can develop due to a variety of acquired causes, with viruses being the most common infectious triggers. Emerging studies on COVID-19 suggest that monocytes and macrophages play a pivotal role in the pathogenesis of this viral infection (5). Cases of SARS-CoV-2 induced secondary HLH during and post-infection have been sparsely reported in children (6, 7). The prevalence of HLH in adult patients with severe COVID-19 is about 7% (8, 9) while there is no clear data on the prevalence of COVID-related HLH in children (10).

Herein, we report the case of a 5-month-old Chinese girl with VEO-IBD caused by a compound heterozygous mutation in IL-10RA, who developed HLH following COVID-19 infection in January 2023. To our knowledge, this is the first documented case of IBD initially presenting as HLH in infancy during the COVID-19 pandemic. The uniqueness of this case lies in the development of HLH following COVID-19, which exacerbated the severity of IBD.

## Case report

A 5-month-old girl was admitted to the Pediatric Intensive Care Unit with a 3-day history of recurrent fever, cough, and ecchymosis, accompanied by diarrhea and significant growth failure. Prior to the onset of symptoms, she had been in contact with her mother, who exhibited clinical manifestations of and was diagnosed with COVID-19. The patient was born at term (40 weeks of gestation) with a birth weight of 3,000 g. Her antenatal, perinatal, and family history was unremarkable, with no significant past medical history, including no prior diagnosis of IBD. Her parents were non-consanguineous. Postnatally, she was discharged in good condition and was exclusively breastfed.

**Figure 1:**
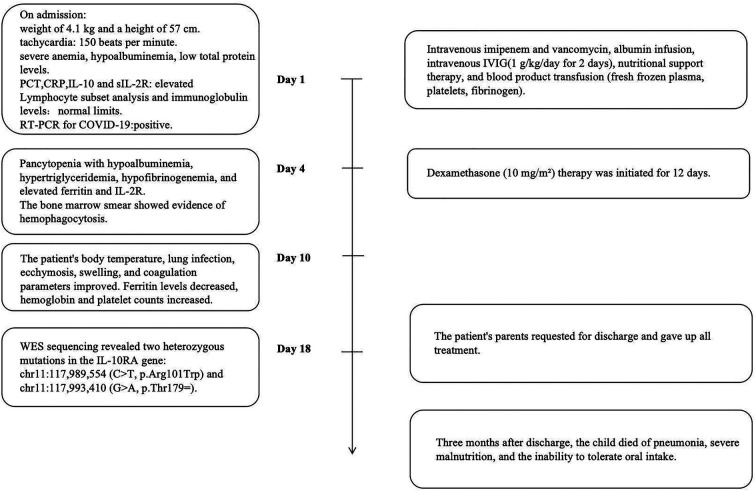
The timeline information of this patient.

The detailed timeline of this patient is shown in Figure 1. On admission, the patient had a weight of 4.1 kg (<3rd percentile) and a height of 57 cm (<3rd percentile). Her initial vital signs indicated tachycardia (heart rate of 150 beats per minute), a temperature of 36.7°C, a respiratory rate of 36 breaths per minute, oxygen saturation of 93% on room air, and blood pressure of 79/50 mmHg in the right upper limb. The patient was conscious but appeared pale and severely malnourished. And the child could babble, track objects with her eyes, and lift her head. Clinical examination revealed generalized ecchymosis, hepatomegaly (liver palpable 6 cm below the right costal margin), anasarca, and a suspected rectovestibular or rectovaginal fistula. There were no signs of oral ulcers, perianal skin tags, joint pain, skin rashes, or lymphadenopathy.

**Figure 2:**
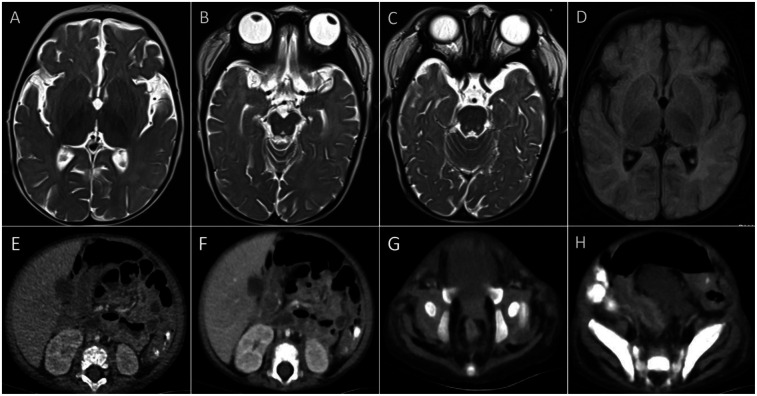
Imagines of CT and MRI. **(A–D)** Brain MRI showed asymmetric signals in the midbrain, pontine, bilateral lateral ventricle triangle and posterior horn. **(E–H)** Abdominal CT revealed thickening and strengthening of the intestinal wall and thickening of the vessels in the colon's hepatocele wall.

Laboratory tests (Table 1) revealed elevated white blood cell counts, severe anemia, hypoalbuminemia, and low total protein levels. Markers of inflammation, including procalcitonin (PCT) and C-reactive protein (CRP), were significantly elevated. Interleukin-10 (IL-10) levels were markedly increased at 427 pg/ml (reference: <9.1 pg/ml), and soluble interleukin-2 receptor (sIL-2R) levels were elevated at 4,375 U/ml (reference: 223–710 U/ml). Kidney function, transaminase, and bilirubin levels were within normal ranges. Lymphocyte subset analysis and immunoglobulin levels, including IgM, IgA, IgG, and IgE, were also within normal limits. Reverse-transcriptase polymerase chain reaction testing for COVID-19 was positive. Additional tests ruled out fungal infections, tuberculosis, Epstein–Barr virus, HIV, adenovirus, parvovirus B19, and hepatitis A, B, and C infections. Blood culture was negative. Cranial imaging with brain MRI revealed asymmetric signal changes in the midbrain, pons, bilateral lateral ventricle trigones, and posterior horns, with restricted diffusion suggestive of a possible genetic metabolic disorder (Figures 2A–D). An echocardiogram was unremarkable, while a chest x-ray at admission indicated pneumonia. Preliminary diagnoses included COVID-19, sepsis, and severe malnutrition. Additionally, due to the combination of severe malnutrition and symmetric changes observed on cranial imaging, a congenital genetic metabolic disorder was strongly suspected. Although preliminary screening with cranial MRI revealed abnormalities, the patient did not experience symptoms such as seizures or changes in consciousness, and due to severe coagulation dysfunction and bleeding tendency, cerebrospinal fluid examination was not performed. For the investigation of genetic metabolic disorder, blood ammonia, lactate, pyruvate, and performed tandem mass spectrometry analysis of blood and urine was tested, all result were negative, which does not support the diagnosis of metabolic encephalopathy, but central nervous system HLH cannot be ruled out.

**Table 1:** Laboratory parameters after chemotherapy.

Laboratory examination	Before (1-day)	After (10-Day)	Reference range
White blood cell (×10^9^/L)	20.3	9.5	4.3–14.2
Neutrophils (%)	62	39.6	7–56
Absolute neutrophil count (×10^9^/L)	2.63	3.76	0.6–7.6
Hemoglobin (g/L)	55	83	97–183
Platelet (×10^9^/L)	68	219	151–536
C-reactive protein (mg/L)	124.7	4.5	0–8
Ferritin (ng/ml)	1,184	615	10–291
prothrombin time (s)	25.2	12.3	7.6–13.6
INR	2.46	1.17	0.8–1.5
APTT(s)	52.4	31.7	18.1–38.1
Fibrinogen (mg/dl)	83	192	200–400
Antithrombin III (%)	35	75	75–125
Total protein (g/L)	35.6	57.7	51–73
Albumin (g/L)	16.6	35.8	38–54
Triglyceride (mmol/L)	3.12	1.95	<1.7

INR, international normalized ratio; APTT, activated partial thromboplastin time.

**Figure 3:**
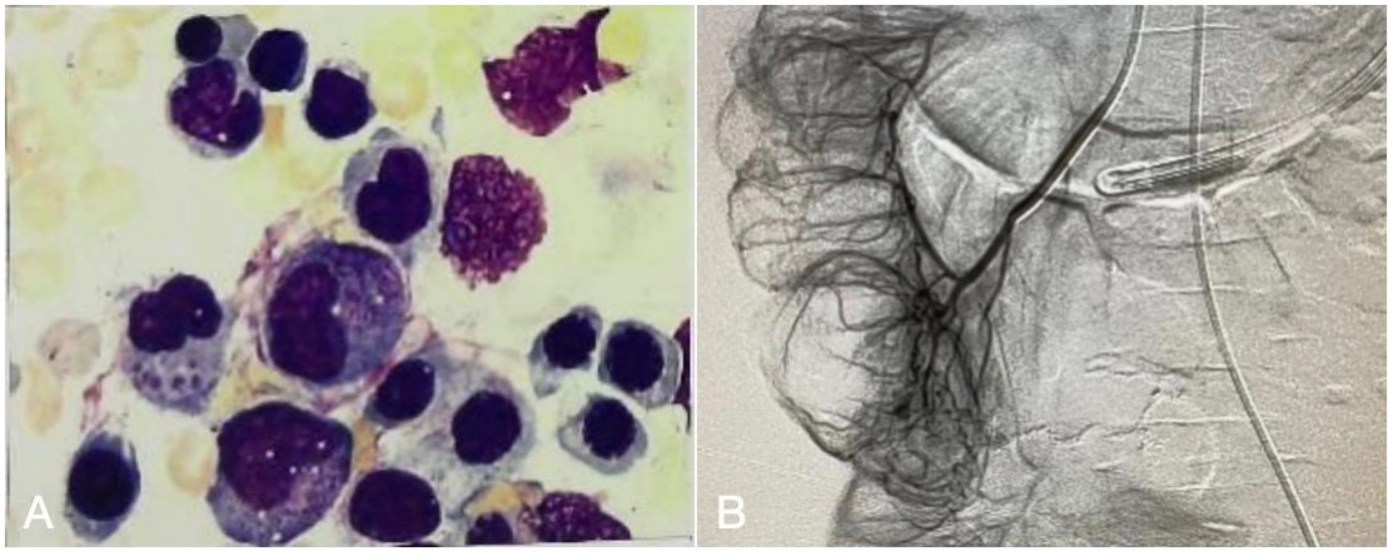
**(A)** Bone marrow smear show hemophagocytic. **(B)** Gastrointestinal angiography and vascular embolization interventional surgery, multiple branch bleeding signs of the inferior mesenteric artery.

**Figure 4:**
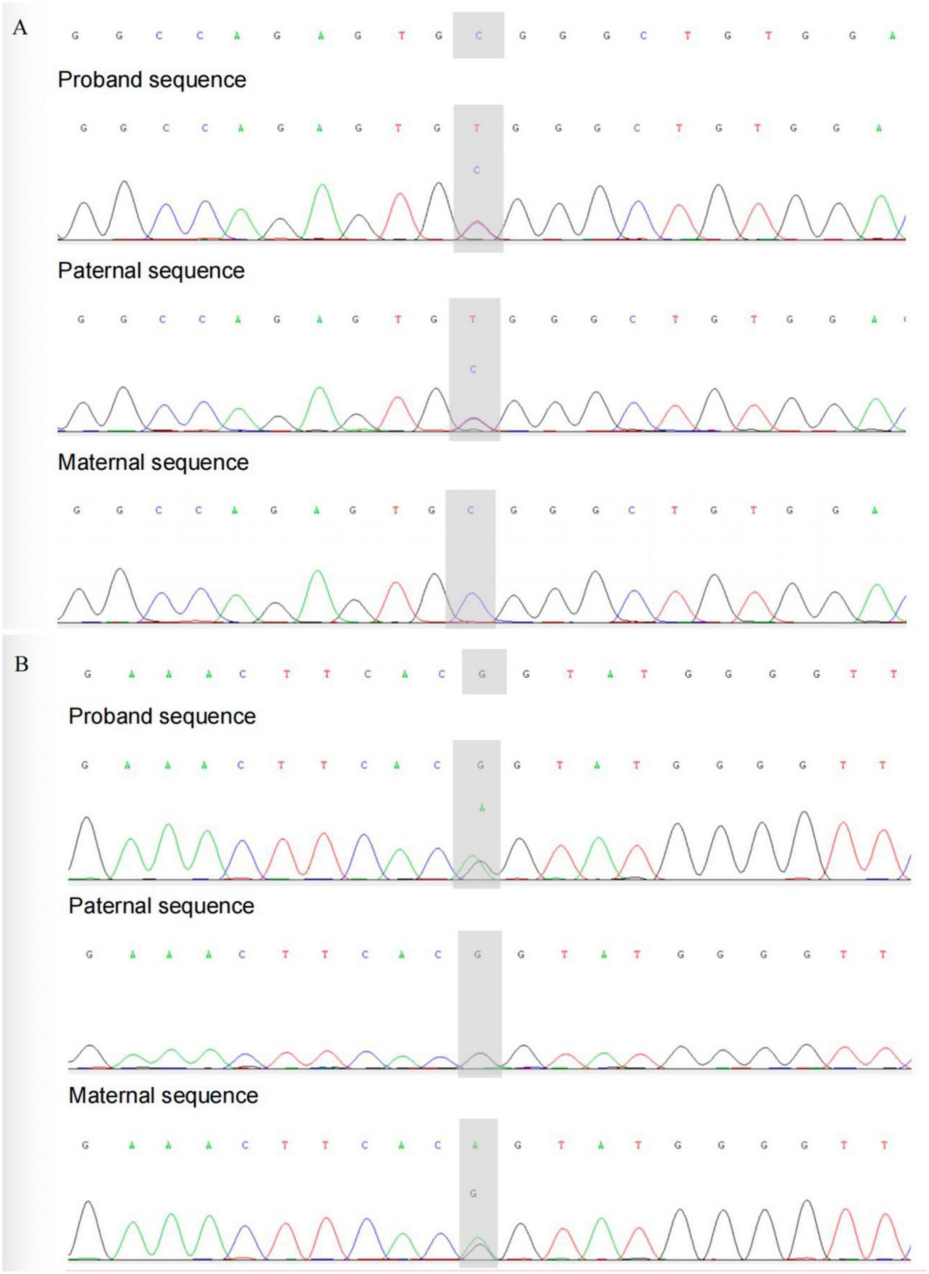
Sanger sequencing validation of this patient and her parents. **(A)** chr11: 117989554. **(B)** chr11: 117993410.

The patient was managed in the PICU with intravenous imipenem and vancomycin, albumin infusion, intravenous IVIG (1 g/kg/day for 2 days), and nutritional support therapy, but the clinical response was limited. Subsequent laboratory investigations revealed pancytopenia with severe neutropenia, anemia, hypoalbuminemia, hypertriglyceridemia, hypofibrinogenemia, and markedly elevated ferritin and IL-2R levels (Table 1). The bone marrow smear showed evidence of hemophagocytosis (Figure 3A). Based on these findings, HLH secondary to COVID-19 infection was suspected, and dexamethasone (10 mg/m^2^ for 12 days) therapy was initiated. Following 6 days treatment, the patient's body temperature, lung infection, ecchymosis, swelling, and coagulation parameters improved (Table 1). Dexamethasone was discontinued due to worsening gastrointestinal bleeding. However, anemia and diarrhea persisted, with ongoing refractory active gastrointestinal bleeding characterized by jam-like stools. Infectious causes of diarrhea were ruled out as stool cultures, parasitic tests, and Clostridium difficile toxin assays were negative. Despite enhanced hemostatic measures, including blood product transfusion (fresh frozen plasma, platelets, fibrinogen), the gastrointestinal bleeding remained uncontrolled. Abdominal computed tomography angiography revealed intestinal wall thickening and vascular engorgement in the colon's hepatic flexure (Figures 2E–H). Gastrointestinal angiography identified multiple bleeding sites in branches of the inferior mesenteric artery, which were successfully embolized during interventional surgery (Figure 3B). No active bleeding was observed following embolization. Given the patient's persistent diarrhea, malnutrition, and the development of HLH in the context of COVID-19, an underlying immunodeficiency or genetic metabolic disorder was suspected. WES sequencing was performed, revealing two heterozygous mutations in the IL-10RA gene: chr11:117,989,554 (C > T, p.Arg101Trp) and chr11:117,993,410 (G > A, p.Thr179=) (Figures 4A,B). The first variant was inherited from the father, while the second was inherited from the mother, consistent with autosomal recessive inheritance. According to the American College of Medical Genetics and Genomics guidelines, both variants were predicted to be pathogenic (PS3 + PM2_Supporting + PM3_very strong for p.Arg101Trp, and PM2_Supporting + PM3_very strong + PP3 for p.Thr179=), with a low population frequency. Based on these genetic findings, the patient was diagnosed with VEO-IBD. Unfortunately, shortly after the interventional procedure, the parents declined further treatment and requested discharge, with a total hospital stay of 18 days. The patient died three months later due to pneumonia, severe malnutrition, and the inability to tolerate oral intake. Endoscopic and colonoscopic evaluations were not possible during hospitalization due to severe active gastrointestinal bleeding.

## Discussion

We present the case of a 5-month-old female infant with signs and symptoms of hyper-inflammation, in whom HLH was initially considered. Genetic testing confirmed IL-10RA mutations (c.301C > T and c.537G > A), diagnostically reclassified from type 28 IBD to IL-10 receptor deficiency per OMIM criteria. To our knowledge, this is the first reported case of IBD in which HLH was initially suspected during the infant period, within the context of the COVID-19 pandemic. The uniqueness of this case lies in the development of HLH following COVID-19 infection, which exacerbated the severity of the IBD and led to recurrent and massive gastrointestinal bleeding. Dissemination of this case report is important as it highlights critical therapeutic, clinical, and management considerations.

In this case report, due to long-term diarrhea, growth and development backwardness, and related manifestations of HLH secondary to COVID-19, such as repeated fever, hepatomegaly, hyperferritinemia, and hypertriglyceridemia, we conducted gene testing to exclude genetic metabolic diseases and immune deficiency diseases. WES identified mutations of the IL-10RA gene, which were determined to be the cause of VEO-IBD. Prior to admission, the patient presented with typical IBD manifestations, such as chronic diarrhea, growth retardation, and malnutrition. She experienced recurrent intestinal bleeding and further weakening of intestinal function. Prolonged antibiotic treatment, coupled with reduced glucocorticoid therapy, further complicated her condition. Additionally, the family's inability to continue supporting her care due to financial constraints contributed to her fatal outcome.

To date, approximately 102 genes related to VEO-IBD have been reported (11), with IL-10R mutations being the most common cause of monogenic IBD in the Han population (12). IL-10 exerts its biological effects by binding to the IL-10R, which subsequently activates Janus kinase 1 and tyrosine kinase 2. This activation leads to the phosphorylation of signal transducer and activator of transcription 3 (STAT3). In turn, STAT3 translocates to the nucleus where it drives the expression of anti-inflammatory mediators (13). Loss-of-function mutations in IL-10RA disrupt the regulation of the IL-6 signaling pathway, leading to an impaired anti-inflammatory response, predominantly in the intestinal tissue, especially within the first months of life (14). Under conditions such as infection, these mutations can result in a hyperactive immune response, characterized by excessive inflammation and tissue damage. This dysregulation is a key pathogenic mechanism underlying type 28 IBD. Of note, an increased probability of SARS-CoV-2 disease is anticipated among patients carrying IL-10R mutations and IL-10 have the capacity to reduce natural killer(NK) cell cytotoxicity (15). Therefore, Viral infections, including COVID-19, often trigger an inappropriate cytotoxic immune response in patients with certain genetic conditions (16), causing uncontrolled activation of T lymphocytes, NK cells, and macrophages, leading to an increase in pro-inflammatory cytokine levels, which can result in HLH. Although many reports currently indicate that HLH can present as the initial symptom of IBD, often occurs after infection, as in our case with COVID-19. However, HLH should also be recognized that it can occur secondary to certain non-infectious diseases or occur after COVID-19 vaccine (17, 18).

It has been shown that IL-10/IL-10R deficiency predominantly presents with treatment-resistant, early-onset IBD within the first months of life (4). Sharifinejad N et al. (19) assessed 286 patients (44.5% female) with IL-10 and/or IL-10R deficiencies who were predominantly from China, more than 93% of patients were <2 years old at the disease onset and were subsequently categorized as infantile-onset IBD. Consanguinity was reported in all evaluable patients with IL-10 deficiency and in 38.2% of patients with IL-10R deficiency. The most prevalent mutations in IL-10RA were c.301C > T (p.R101W) and c.537G > A (p.T179 T). Defects in IL-10 signaling are associated with extra-intestinal inflammation, severe infections, and the development of immune diseases (20). Patients with IL-10R deficiency had a high frequency of dermatologic manifestations and failure to thrive. An increased susceptibility for B cell lymphoma has also been reported in patients with IL-10/IL-10R deficiency (21). However, there is no clear genotype-phenotype correlation in IL-10/IL-10R deficiency according to the previous cohort studies (22). In Chinese children, symptoms such as diarrhea, fever, oral ulcers, bleeding, and perianal lesions are common. However, younger children who cannot express symptoms like abdominal pain may present with weight loss and fatigue when affected. Children with VEO-IBD generally have more severe clinical symptoms, more extensive intestinal inflammation, higher rates of treatment resistance, and rapid disease progression compared to adults. Diagnosing IBD can be particularly challenging in infants, as its symptoms overlap with those of more common conditions such as milk allergy, infections, and lymphoid follicular hyperplasia (22).

In our case, the underlying condition of IBD made the patient more susceptible to COVID-19 infection, which in turn triggered HLH. The patient presented with the typical triad of abnormalities, including hypertriglyceridemia, hypofibrinogenemia, and hyperferritinemia. Bone marrow smear revealed hemophagocytic, along with fever, cytopenia, hepatosplenomegaly, and elevated ferritin levels, fulfilling the diagnostic criteria for HLH (≥5 criteria). This suggested HLH as the final diagnosis. Therefore, genetic testing was performed to rule out genetic metabolic diseases and immunodeficiency diseases, ultimately leading to the discovery of IBD as the underlying condition. HLH is thankfully a rare condition, it can be mistaken for sepsis or IBD exacerbation due to overlapping clinical characteristics. However, our patient developed a viral infection without the use of immunosuppressants. We hypothesize that excessive immune activation and cytokine storm resulting from the combination of IBD, COVID-19 infection, and HLH contributed to more extensive systemic damage. This suggests that both HLH and COVID-19 are hyperinflammatory syndromes characterized by hypercytokinemia and share similar pathophysiological mechanisms. It highlights the potential for more serious consequences when IBD coexists with HLH during a COVID-19 infection.

The youngest reported cases of HLH triggered by COVID-19 infection were neonates, only 6 weeks old (14), all of whom were infected by their mothers. Since HLH is associated with a high mortality rate, early identification is critical for timely treatment and better prognosis. Intervention is essential to avoid irreversible organ-specific damage. HLH is typically treated with immunosuppressive therapy to induce remission (etoposide in combination with dexamethasone). For patients with primary HLH, allogeneic hematopoietic stem cell transplantation (allo-HSCT) is considered once the high-inflammatory state is controlled. Besides, cytokine-targeted therapy and gene therapy can be a treatment option (23). Our patient was treated with both the HLH-2004 chemotherapy protocol and COVID-19 therapy as recommended by the guidelines, resulting in improvements in clinical manifestations and laboratory findings. For children with VEO-IBD type 28 caused by IL-10RA gene variation, traditional treatment methods such as glucocorticoids and immunosuppressants often yield poor results. Currently, allo-HSCT is one of the most effective treatments for VEO-IBD patients with IL-10 signaling deficiency (24). However, the patient's condition deteriorated due to the family's refusal to continue treatment, and she ultimately died within a few months.

The limitations of this study include the lack of gastrointestinal endoscopy, which would have allowed assessment of the intestinal mucosal condition, as well as the absence of follow-up treatment data. The short period between diagnosis and death prevented the evaluation of the efficacy of immunotherapy for IBD in the context of HLH.

## Conclusion

We report a case of a Chinese pediatric patient with VEO-IBD, complicated by HLH following a COVID-19 infection, with an IL-10RA gene mutation. The correlation between IBD and HLH emphasizes the importance of early recognition and heightened awareness among clinicians, particularly in patients with underlying autoimmune diseases or concurrent COVID-19 infections. These conditions can exacerbate the severity of IBD and influence its progression; therefore, close monitoring and prompt treatment are essential.

## Data Availability

The original contributions presented in the study are included in the article/Supplementary Material, further inquiries can be directed to the corresponding authors.
